# Extended Archaeal Histone-Based Chromatin Structure Regulates Global Gene Expression in *Thermococcus kodakarensis*

**DOI:** 10.3389/fmicb.2021.681150

**Published:** 2021-05-13

**Authors:** Travis J. Sanders, Fahad Ullah, Alexandra M. Gehring, Brett W. Burkhart, Robert L. Vickerman, Sudili Fernando, Andrew F. Gardner, Asa Ben-Hur, Thomas J. Santangelo

**Affiliations:** ^1^Department of Biochemistry and Molecular Biology, Colorado State University, Fort Collins, CO, United States; ^2^Department of Computer Science, Colorado State University, Fort Collins, CO, United States; ^3^Molecular Enzymology Division, New England Biolabs, Inc., Ipswich, MA, United States

**Keywords:** archaea, histone, chromatin, transcriptome, *Thermococcus*, RNA-seq

## Abstract

Histone proteins compact and organize DNA resulting in a dynamic chromatin architecture impacting DNA accessibility and ultimately gene expression. Eukaryotic chromatin landscapes are structured through histone protein variants, epigenetic marks, the activities of chromatin-remodeling complexes, and post-translational modification of histone proteins. In most Archaea, histone-based chromatin structure is dominated by the helical polymerization of histone proteins wrapping DNA into a repetitive and closely gyred configuration. The formation of the archaeal-histone chromatin-superhelix is a regulatory force of adaptive gene expression and is likely critical for regulation of gene expression in all histone-encoding Archaea. Single amino acid substitutions in archaeal histones that block formation of tightly packed chromatin structures have profound effects on cellular fitness, but the underlying gene expression changes resultant from an altered chromatin landscape have not been resolved. Using the model organism *Thermococcus kodakarensis*, we genetically alter the chromatin landscape and quantify the resultant changes in gene expression, including unanticipated and significant impacts on provirus transcription. Global transcriptome changes resultant from varying chromatin landscapes reveal the regulatory importance of higher-order histone-based chromatin architectures in regulating archaeal gene expression.

## Introduction

**FIGURE 1:**
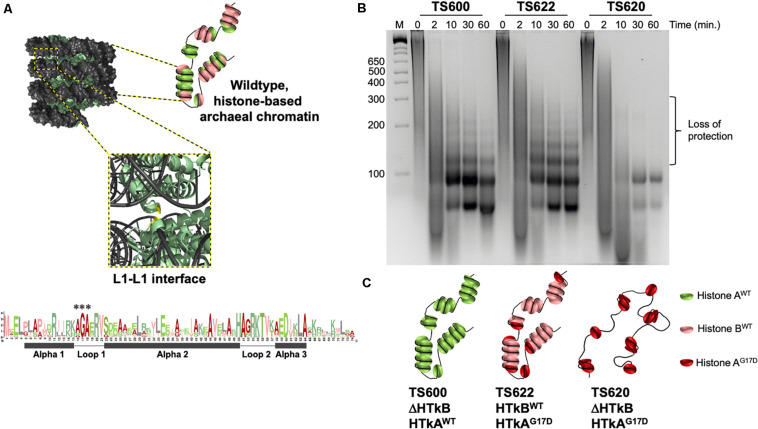
A single wild-type histone protein is sufficient for normal DNA protection in *T. kodakarensis*. **(A)** Diagrammatic representation of wildtype chromatin modeled from the archaeal histone-based chromatin crystal structure: 9 polymerized histone B dimers (pale green) wrapped by DNA (gray) adapted from Mattiroli et al. (2017). The central glycine in the AGA motif at the Loop1–Loop1 interface is colored in red. A Logo-plot highlights the conservation of this motif. Histone dimers may be heterogeneously composed. **(B)** DNA fragments resulting from micrococcal nuclease (MNase) digested chromatin demonstrate the state of chromatin structure in TS600, TS622, and TS620. Chromatin purified from TS600 (TK1413^*WT*^:histone A/ΔTK2289:histone B) resists MNase digestion over time, resulting in a laddered DNA banding pattern. Prominent 60 and 90 bp bands in addition to higher molecular weight bands (increasing 30 bp increments up to ∼300 bp) represent varying levels of histone dimerization and MNase protection. Chromatin purified from TS622 (TK1413^*G*17*D*^:histone A/TK2289^*WT*^:histone B) exhibits an identical protection pattern to TS600 despite encoding a variant (G17D) histone A. This suggests a single WT histone is sufficient for normal chromatin structure formation. Chromatin purified from TS620 (TK1413^*G*17*D*^:histone A/ΔTK2289:histone B) exhibits a markedly different protection pattern from TS600 and TS622. The presence of only a variant (G17D) histone A results in a loss of DNAs protected above 90 bp, demonstrating the disruption of the L1–L1 interface interferes with continued histone dimer polymerization. **(C)** Diagrammatic representation of the potential chromatin structures in TS600, TS622, and TS620.

Most Archaea encode histone proteins to organize their DNA into a protein:DNA complex known as chromatin (Sandman and Reeve, 2000, 2001, 2006; Peeters et al., 2015; Mattiroli et al., 2017; Bhattacharyya et al., 2018; Henneman et al., 2018; Sanders et al., 2019b; Henneman et al., 2020; Stevens et al., 2020; Bowerman et al., 2021; Laursen et al., 2021). The organization and tractability of chromatin structure is essential to regulate adaptive gene expression (Wilkinson et al., 2010; Nalabothula et al., 2013; Mattiroli et al., 2017). Archaeal and eukaryotic histone proteins are highly homologous, sharing conserved residues that contact DNA and retain structural elements of the histone fold (Sandman and Reeve, 2000; Soares et al., 2000; Mattiroli et al., 2017). In most cases, however, the extensions or tails common to eukaryotic histones are not present in archaeal histone isoforms (Peeters et al., 2015; Mattiroli et al., 2017; Nishida and Oshima, 2017; Bhattacharyya et al., 2018; Henneman et al., 2018; Henneman et al., 2020; Sanders et al., 2019b; Stevens et al., 2020). Unlike their eukaryotic counterparts, archaeal histones can homodimerize and spontaneously oligomerize to form chromatin with a single protein (Decanniere et al., 2000; Mattiroli et al., 2017; Bhattacharyya et al., 2018; Henneman et al., 2020; Stevens et al., 2020; Bowerman et al., 2021; Laursen et al., 2021), and thus unlike their eukaryotic counterparts (Luger et al., 1997), archaeal chromatin structures are not defined in size. Continued polymerization of archaeal histone proteins produces a symmetrical superstructure composed of increasing lengths of DNA wrapped around a core of polymerized histone dimers (Mattiroli et al., 2017). This extended histone-based chromatin structure, alternatively termed the hypernucleosome (Bhattacharyya et al., 2018; Henneman et al., 2018; Henneman et al., 2020), is the biological form of archaeal chromatin within which gene expression is regulated (Figure 1A).

The polymerization of histone dimers in Archaea does not resemble the nucleosome-nucleosome interactions observed in eukaryotes (Luger et al., 1997; Mattiroli et al., 2017; Sanders et al., 2019b; Henneman et al., 2020; Stevens et al., 2020; Bowerman et al., 2021). Alignments and analyses of unique archaeal histone sequences reveals the vast majority of archaeal histone proteins retain only the residues comprising the core eukaryotic histone-fold and that many amino acids are highly conserved in positions that form the histone-fold or mediate DNA interactions (Mattiroli et al., 2017; Nishida and Oshima, 2017; Zaremba-Niedzwiedzka et al., 2017; Henneman et al., 2020; Stevens et al., 2020). The core histone fold, common to eukaryotic and archaeal histones, consists of three alpha helices (α1, α2, and α3) connected through two flexible loops (L1 and L2). A conserved alanine-glycine-alanine (AGA) motif in L1 was identified that was not obviously involved in protein-DNA interactions or in supporting the core histone-fold (Mattiroli et al., 2017; Bhattacharyya et al., 2018; Henneman et al., 2018; Henneman et al., 2020; Stevens et al., 2020) (Figure 1B). The crystal structure of archaeal histone-based chromatin provided the first clues to the importance of the highly conserved motif in L1, wherein the small side chains of these nearly invariant amino acids permitted the close proximity of adjacent gyres of DNA within the superhelix at the Loop1–Loop1 (L1–L1) interface. The conservation of these residues was thus hypothesized to support the biologically important, tight-packing of archaeal histone-based chromatin architectures (Mattiroli et al., 2017; Bhattacharyya et al., 2018; Henneman et al., 2018; Henneman et al., 2020; Stevens et al., 2020).

Archaeal histone-based chromatin digestion patterns reveal the existence of histone-DNA superstructures that vary in length from protection of ∼60 bp of DNA by a histone tetramer to protection of ∼300 bp of DNA by a semi-continuous polymer of ∼10 symmetrically bound histone dimers. Given the different lengths and proposed dynamic structures of archaeal histone-based chromatin, we adopt the term archaeal histone-based chromatin polymers (AHCPs) to denote histone:DNA superstructures wherein each additional histone dimer can wrap ∼30 bp of DNA to extend the AHCP (Nalabothula et al., 2013; Mattiroli et al., 2017; Bhattacharyya et al., 2018; Sanders et al., 2019a; Bowerman et al., 2021). While dynamic changes in AHCPs are expected, particularly in species with multiple histone isoforms (Henneman et al., 2020; Stevens et al., 2020), it is also likely that specific DNA sequences and loci are more likely to retain extended AHCPs that are important for regulating gene expression (Čuboňová et al., 2012; Nalabothula et al., 2013; Dulmage et al., 2015). Although the presence of several histone isoforms in many Archaea suggests the potential for variation in superhelix composition and length (Mattiroli et al., 2017; Bhattacharyya et al., 2018; Henneman et al., 2018; Sanders et al., 2019b; Henneman et al., 2020; Stevens et al., 2020; Bowerman et al., 2021), the viability of strains with only a single histone demonstrate that even homopolymers permit formation of AHCPs *in vivo* (Sandman and Reeve, 2001, 2006; Santangelo et al., 2009; Čuboňová et al., 2012; Dulmage et al., 2015). The biological importance of AHCPs is supported by the evolutionary retention of the AGA motif in L1 which permits close association of adjacent superhelical gyres. Importantly, allelic substitutions of the central glycine within the L1-AGA motif abrogated formation of extended AHCPs, decreased cellular fitness and resulted in the loss of adaptive gene expression when actively growing cultures were moved to a new metabolic environment (Jäger et al., 2014; Mattiroli et al., 2017).

The importance of AHCPs in modulating gene expression suggests unique archaeal gene regulation strategies that take advantage of mechanisms to retain or abolish extended archaeal histone-based chromatin structures (Sanders et al., 2019b; Stevens et al., 2020; Bowerman et al., 2021). To evaluate the normal contribution of AHCPs to gene regulation, we generated strains of the model archaeon *Thermococcus kodakarensis* (Farkas et al., 2013; Gehring et al., 2017; Atomi and Reeve, 2019) wherein genomically encoded histone variants impacted global genomic architecture and quantified the gene expression changes resultant from modified AHCP architectures. *T. kodakarensis* normally encodes two closely related histone isoforms, termed Histone A (HTkA) and Histone B (HTkB), but strains encoding only a single histone variant are viable (Čuboňová et al., 2012; Mattiroli et al., 2017; Sanders et al., 2019b). To evaluate the consequences of altering AHCPs in archaeal cells, strains of *T. kodakarensis* were constructed to encode only HTkA in WT form (HTkA^*WT*^; ΔHTkB), only HTkA with a single amino acid substitution G17D (HTkA^*G*17*D*^; ΔHTkB), or retain HTkB in WT form and express HTkA^*G*17*D*^ (HTkA^*G*17*D*^; ΔHTkB^*WT*^). Substitution of just a single residue, G17, is sufficient to disrupt AHCP formation *in vivo* beyond ∼90 bp by inhibiting the close association of adjacent gyres of AHCPs (Bhattacharyya et al., 2018). We report here that retention of a single WT histone variant is sufficient to maintain extended AHCPs in archaeal cells, but extended AHCPs are abolished in strains encoding only the mutated AGA motif, HTkA^*G*17*D*^.

We demonstrate here, using comparative differential RNA-seq analyses of strains with unique AHCP landscapes, that substantial and genome-wide variations in gene expression result from alternating archaeal histone-based chromatin structures, underscoring the importance of AHCPs in normal regulation of gene expression. Expression differences observed in strains lacking extended AHCPs suggest architectural changes in AHCPs are most impactful for the proper regulated expression of chemotaxis-, motility- and proviral-encoding regions of the genome. The global regulatory potential of AHCPs is confirmed and offers impactful routes to control archaeal gene expression by modulating chromatin architectures and AHCP formation *in vivo*.

## Materials and Methods

### Strain Construction and Growth Conditions

*Thermococcus kodakarensis* strains were constructed essentially as previously described (Gehring et al., 2017). While *T. kodakarensis* strains deleted for either TK2289 or TK1413 remain viable, strains deleted for TK1413 are no longer genetically accessible (Čuboňová et al., 2012). Thus, to generate single histone-encoding strains with substitutions that impact AHCP formation, we first deleted TK2289, then introduced allelic changes to TK1413. *T. kodakarensis* strain TS600 was constructed from parental strain TS559 by markerless deletion of TK2289 (HTkB). Strain TS622 was constructed from TS600 by allelic substitution of TK1413 (HTkA) for TK1413^*G*17*D*^ (HTkA^*G*17*D*^). TS620 was constructed by markerless deletion of TK2289 (HTkB) from TS622. Allelic substitution of TK1413 for TK1413^*G*17*D*^ was confirmed by PCR amplification of the TK1413 loci and subsequent sequencing while deletion of TK2289 was confirmed via diagnostic PCR with primers flanking TK2289. Cultures were grown at 85°C in artificial seawater (ASW) supplemented with 0.5% (w/v) tryptone, 0.5% (w/v) yeast extract (ASW-YT), trace mineral solution and vitamin mixture (nutrient rich medium). ASW contains, per l, 20 g NaCl, 3 g MgCl_2_⋅6H_2_O, 6 g MgSO_4_⋅7H_2_O, 1 g (NH_4_)_2_SO_4_, 200 mg NaHCO_3_, 300 mg CaCl_2_⋅2H_2_O, 0.5 g KCl, 420 mg KH_2_PO_4_, 50 mg NaBr, 20 mg SrCl_2_⋅6H_2_O, and 10 mg Fe(NH_4_)_2_(SO_4_)2⋅6H_2_O. The trace mineral solution (1,000×) contains, per l, 0.5 g MnSO_4_ 6H_2_O, 0.1 g CoCl_2_⋅6H_2_O, 0.1 g ZnSO_4_⋅7H_2_O, 0.01 g CuSO_4_⋅5H_2_O, 0.01 g AlK(SO_4_)_2_⋅12H_2_O, 0.01 g H_3_BO_3_, and 0.01 g Na_2_MoO_4_⋅2H_2_O. The vitamin mixture (200×) contains, per l, 0.2 g niacin, 0.08 g biotin, 0.2 g pantothenate, 0.2 g lipoic acid, 0.08 g folic acid, 0.2 g p-aminobenzoic acid, 0.2 g thiamine, 0.2 g riboflavin, 0.2 g pyridoxine, and 0.2 g cobalamin. When present, sodium pyruvate was added at 5 g per l, agmatine sulfate to 1 mM and 6- methyl purine (6MP) to 100 μM (ASW-YT-Pyr). Elemental sulfur (S∘) was added at 2 g per l in liquid media (ASW-YT-) but was replaced by polysulfides in solid media. Polysulfide solution (500×) contained, per l, 66.7 g sodium sulfide (Na_2_S⋅9H_2_O) and 3 g sulfur. Gelrite was added to 1% (w/v) to solidify media.

### Chromatin Isolation and Micrococcal Nuclease Digestion

Chromatin isolation and micrococcal nuclease digestions were adapted from Mattiroli et al. (2017). Strains TS600, TS620 and TS622 were individual grown to an optical density (measured at 600 nm) of ∼0.5 in liquid ASW-YT-S and each starter culture was used to inoculate (1:100) 200 ml of ASW-YT-Pyr cultures that were allowed to grow to an optical density (measured at 600 nm) of ∼0.5 to encourage chromatin reprograming. Cultures were pelleted at ∼8,000 × *g* and immediately frozen at −80°C. Cell pellets were resuspended in 1.0 mL of MNase buffer (50 mM Tris-HCl pH 8.0, 100 mM NaCl, and 1 mM CaCl_2_) per 0.2 g of cell mass and ground to homogeneity with a mortar and pestle. Homogenized cells were mechanically lysed by repeated liquid nitrogen freezing and subsequent grinding five times. Whole cell lysate was gently clarified at 1,700 × *g* for 5 min and the chromatin containing clarified lysate was RNase A digested (Sigma, 4,000 U) for 1 h at 37°C. ∼1,500 U of micrococcal nuclease (New England Biolabs) was added to chromatin and aliquots (∼100 μl) of digested DNAs were extracted by the addition of 300 μl of 10 mM Tris-HCl pH 8.0 and 400 μl of phenol/chloroform/isoamyl alcohol (25:24:1). Following thorough emulsion and centrifugation at 10,000 × *g* for 5 min, ∼200 μL of the DNA-containing aqueous layer was precipitated by the addition of an equal volume of 1 M Tris-HCl pH 8.0 and 2.6 volumes of 100% ethanol proceeding a 1-h incubation at −80°C. DNAs were pelleted in a 4°C centrifuge at 10,000 × g for 30 min and subsequently resolved in a 4% agarose gel.

### RNA Isolation

TS600 and TS622 were grown in triplicate to an optical density (measured at 600 nm) of ∼0.5 in liquid ASW-YT-S and used to inoculate (1:100) 300 ml of ASW-YT-Pyr per strain and allowed to grow to an optical density (measured at 600 nm) of ∼0.05 to encourage chromatin reprograming. Cultures were rapidly chilled and pelleted at 8,000 × g for 5 min and then resuspended in 1.0 mL of Trizol (Invitrogen) with a 10-min incubation at room temperature. A total of 200 μl of chloroform was added followed by centrifugation at 10,000 × *g* at 4°C for 15 min yielding an RNA-containing aqueous layer which was added to 500 μl of isopropanol and incubated at room temperature for 10 min. Centrifugation at 10,000 × *g* for 15 min at 4°C produced an RNA pellet that was washed with 1 ml of 75% ethanol and subsequently resuspended in 88 μl of RNase-free H_2_O, 10 μl of DNaseI buffer, and 1 μl of DNaseI (New England Biolabs) to digest residual DNA (37°C for 30 min). Replicate samples were prepared identically.

### RNA-Seq Library Preparation

#### TS600 and TS622

Using ∼120 ng of RNA that was depleted of rRNA, following the NEBNext^®^ rRNA Depletion Kit (E6310), cDNA libraries were constructed with New England Biolabs NEBNext^®^ Ultra^TM^ Directional RNA Library Prep kit for Illumina^®^ (E7420s) and NEBNext^®^ Multiplex Oligos for Illumina^®^ (Index primer set 1, E7335) according to the manufacturer’s procedure. The multiplexed libraries were sequenced at Cofactor Genomics using one high output NextSeq Illumina^®^ run for single-end reads with a minimum read length of 75 bp and with a requested >40 million reads per sample.

#### TS600 and TS620

A total of 1.5 μg of RNA was processed at Novogene for Prokaryotic RNA-seq, specifically for rRNA depletion (Ribo-Zero^TM^ Magnetic Kit), library construction (NEBNext^®^ Ultra^TM^ RNA Library Prep kit) and 150 bp paired-end sequencing on a HiSeq Illumina^®^ platform.

### Data Preprocessing

#### TS600 and TS622

RNA-Seq reads were first analyzed for quality control using FastQC (Andrews, 2010). To remove adapter sequences and other artifacts, fastx-trimmer was used to trim the first 11 positions in each read (Gordon). After filtering, the reads were aligned to the *T. kodakarensis* (KOD1) reference genome using bowtie (Langmead, 2010) with the parameter *-m 1*, thus ensuring suppression of all multiply aligned reads. Finally, the bowtie output was converted to BAM format, sorted, and indexed using samtools (Li et al., 2009).

#### TS600 and TS620

RNA-Seq reads were first analyzed for quality control using FastQC (Andrews, 2010). To remove adapter sequences and other artifacts, fastx-trimmer was used to trim the first 19 positions in each read (Gordon). After filtering, the reads were aligned to the *T. kodakarensis* (KOD1) reference genome using bowtie2 (Langmead and Salzberg, 2012) with default parameters. Next, in each library, reads that aligned to multiple locations were filtered out. Finally, the filtered output was converted to BAM format, sorted, and indexed using samtools (Li et al., 2009).

### Differential Gene Expression Analysis

To identify differentially expressed genes we used EdgeR (Robinson et al., 2009). Read counts for every gene in the Ensembl annotations of the species were generated using a custom python script that used SpliceGrapher (Rogers et al., 2012) and pysam (Heger et al., 2014). The EdgeR *p*-values were adjusted for multiple comparisons using the Benjamini-Hochberg method (Benjamini and Hochberg, 1995). Finally, we used a cutoff of 1.00 on the transcript abundance (log CPM) and fold change (log FC).

### Data Visualization and Plotting

To visualize differential gene expression, we used Plotly (Sievert et al., 2017) to generate the MA plots. All analyses are provided in the supplementary jupyter notebooks. Circos (Krzywinski et al., 2009) plots were generated using the FPKM values of genes, measured using stringtie2 tool (Pertea et al., 2015) using the Ensembl gene annotations.

### DNA Sequencing of TS620

Genomic DNA was purified from strain TS620 using the Monarch Genomic DNA Purification kit (New England Biolabs). Pacific Biosciences (PacBio) libraries were constructed following the Pacific Biosciences Template Preparation and Sequencing Protocol. The library was then sequenced on a PacBio Sequel instrument using Polymerase 3.0 Chemistry and diffusion loading for 600 min. The data was analyzed using PacBio SMRT Analysis tools.

## Results

### A Single Histone Protein Is Sufficient for AHCP Formation

To assess the role of chromatin superstructure, reflected by the totality of AHCPs, on gene expression in living cells, we generated *T. kodakarensis* strains that encode histone variants known to impact AHCP length. While strains retaining only a single histone isoform are viable, reducing the concentration of total histone proteins (Čuboňová et al., 2012; Mattiroli et al., 2017; Sanders et al., 2019b), or retaining histone variants with dramatically reduced DNA binding affinity (Čuboňová et al., 2012; Mattiroli et al., 2017) is not tolerated in *T. kodakarensis*. We thus carefully constructed strains wherein we retained the natural expression profile of one or both histone proteins but altered the sequence of individual histone-encoding genes to produce proteins at native levels (Mattiroli et al., 2017) that impact AHCP formation. Strains encoding only a single histone isoform in WT or variant form (Supplementary Figure S1) were generated, as were strains wherein one isoform remained WT, and the other was modified to replace G17 with D. Markerless modification or deletion of the genes encoding the endogenous histone proteins, HTkA (TK1413) and HTkB (TK2289), in their natural context allowed for preservation of the native promoter elements and did not impact the surrounding loci. Strain TS559 served as the parental strain for construction of three strains with varied histone compositions. Deletion of TK2289 (encoding HTkB) resulted in strain TS600 (encoding only HTkA^*WT*^). Modification of TK1413 to generate a G17D variant of HTkA in an otherwise native background, including the presense of WT HTkB, generated strain TS622. TS620 combines both genetic modifications, thereby generating a strain supported by only the synthesis of a single, G17D HTkA variant. Deletion of TK2289, encoding HTkB, was confirmed in TS600 and TS620 by PCR amplification of the flanking genomic regions and sequencing to confirm the exact desired endpoints of the genomic modification (Supplementary Figure S1B). HTkA^*G*17*D*^ variants were confirmed in TS620 and TS622 by amplifying the entire HTkA coding and promoter sequences followed by Sanger sequencing to confirm retention of all native regulatory and coding sequences with the exception of the desired missense mutation (Supplementary Figures S1B,C).

To assess the impacts of varied histone isoforms on AHCP formation, total chromatin purified from strains TS600, TS620, and TS622 was subjected to micrococcal nuclease (MNase) digestion. MNase digestion provides a rapid, genome-wide mechanism to define minimal units of chromatin - such as the nucleosome in Eukarya. Digestion of chromatin isolated from TS600 confirmed the previously observed wildtype AHCP protection patterning – a prominent ∼60 bp DNA fragment along with larger DNA fragments in increasing ∼30 bp increments (up to ∼300 bp) – indicative of varied-length AHCPs (Čuboňová et al., 2012; Mattiroli et al., 2017; Henneman et al., 2018; Rojec et al., 2019; Henneman et al., 2020; Stevens et al., 2020) (Figures 1B,C). The persistence of the distinct 60 + 30(n) ladder demonstrates that AHCPs are varied in length but generally stable, thus providing architectures that can be exploited to regulate DNA accessibility and gene expression. TS600 encodes only the HTkA isoform, therefore all histone-based chromatin structure in this strain is composed of this single histone (Figure 1C).

In contrast, when HTkA was modified to place a larger and charged residue within the AGA motif of L1 (G17D), the chromatin from strain TS620 displayed a dramatically different MNase protection pattern (Figure 1B). Discrete DNA fragments >90 bp were absent, demonstrating histone:DNA interactions occurred to allow initial DNA wrapping but that continued polymerization to form extended AHCPs was not possible. The observed digestion pattern is consistent with previous (Mattiroli et al., 2017) digestions of chromatin from variant HTkA^*G*17^-encoding strains, suggesting histone dimers form tetramers, protecting ∼60 bp of DNA, and an additional dimer interacts to form a hexamer, protecting ∼90 bp of DNA, but that larger associations of histone dimers are restricted due to clashes between adjacent gyres of AHCPs and loss of potential electrostatic interactions across the adjacent gyres (Henneman et al., 2018; Henneman et al., 2020; Bowerman et al., 2021). Thus, across the entire genome, the single HTkA^*G*17*D*^ variant encoded in TS620 disrupts the L1–L1 interface within AHCPs, preventing continued polymerization of histone dimers that normally provides a route to extended AHCP formation.

Surprisingly, digestion of total chromatin purified from TS622 resulted in an MNase protection pattern nearly identical to TS600 (Figure 1B) suggesting the presence of variant HTkA^*G*17*D*^ did not interfere with superhelix formation of the HTkB isoform. Given that chromatin from strain TS620 demonstrates HTkA^*G*17*D*^ alone cannot make up the superhelix, it is likely that all the larger regions of DNA protection in strain TS622 result from superstructures formed entirely of HTkB. It is possible that at least some of the smaller (60–90 bp) protected DNA fragments result from chromatin structures composed entirely of HTkA^*G*17*D*^ or from HTkB/HtkA^*G*17*D*^ heteromers; additionally, it is possible HTkA^*G*17*D*^ may cap or terminate the superhelices composed of HTkB resulting in protection of >90 bp fragments (Henneman et al., 2020; Stevens et al., 2020) (Figures 1B,C).

### AHCP Structures Regulate Genome-Wide Gene Expression

With histone composition altered in strain TS622, and histone-composition and extended AHCP formation disrupted in strain TS620, we sought to quantify the transcriptomes of each strain in response to an environmental shift (Jäger et al., 2014; Atomi and Reeve, 2019). Environmentally cued changes to histone-based chromatin architecture are a known mechanism to regulate gene expression, and for the *Thermococcales*, one of the largest determinants of metabolism and gene expression profiles is the availability of different terminal electron acceptors (Jäger et al., 2014; Mattiroli et al., 2017). Strains with variant histone- and AHCP-landscapes were grown to early exponential phase under conditions that permit elemental sulfur (S∘) to serve as the terminal-electron acceptor, then rapidly transferred to conditions wherein S∘ was absent, signaling a metabolic shift requiring substantial changes in gene expression for continued rapid growth.

**FIGURE 2:**
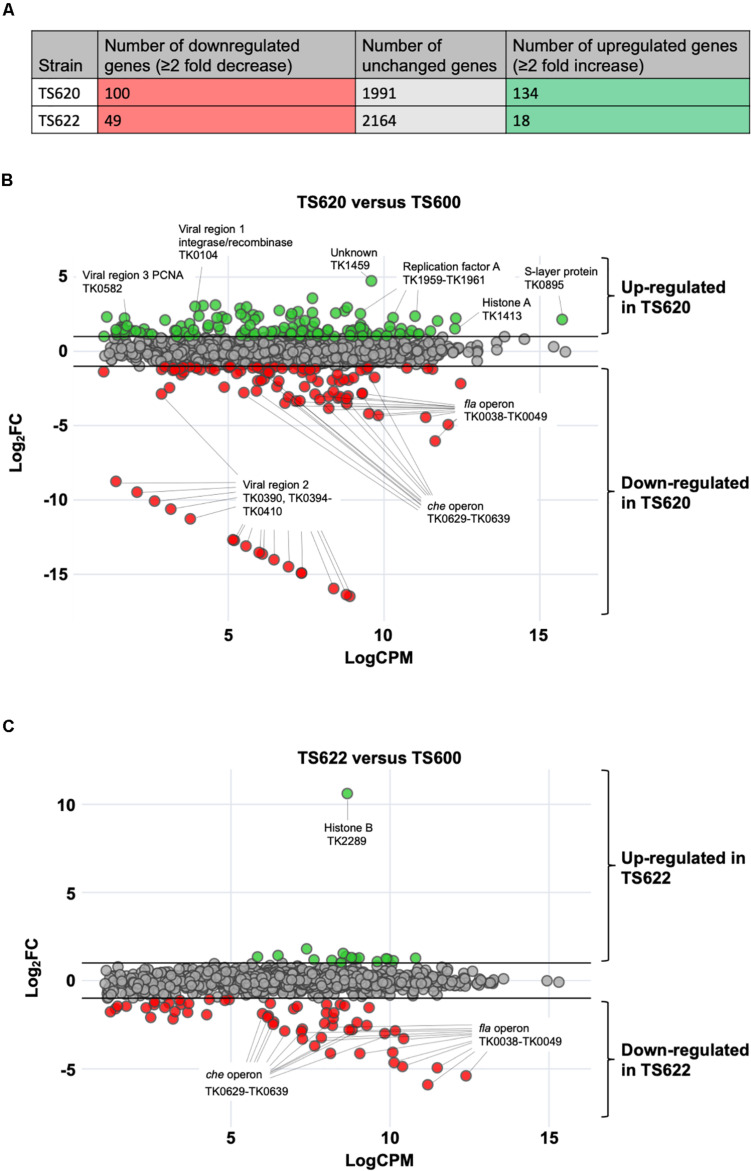
Altering 3-dimensional chromatin structure dramatically alters gene expression in *T. kodakarensis*. **(A)** In TS620 (compared to TS600) 100 protein-coding genes were downregulated while 134 protein-coding genes were upregulated. In TS622 (compared to TS600), 49 protein-coding genes were downregulated while 18 protein-coding genes were upregulated. **(B)** In TS620 (TK1413^*G*17*D*^:histone A/ΔTK2289:histone B) differential RNA-sequencing, represented in an MA plot, revealed a number of genes are significantly upregulated and downregulated when compared to TS600 (TK1413^*WT*^:histone A/ΔTK2289:histone B). Green transcripts are significantly (≥2 fold change) enriched in TS620 when compared to TS600. Red transcripts are significantly depleted (≥2 fold change) in TS620 when compared to TS600. Notably, in TS620, a number of single stranded binding proteins (TK1959-1961: replication protein A subunits Rpa32, Rpa14, and Rpa41) are significantly enriched in TS620. TK1413^*G*17*D*^ was found to be enriched in TS620 when compared to TK1413^*WT*^ in TS600. In TS620 transcripts involved in cell motility and cell signaling (TK0038-TK0049: archaeal Fla operon and archaeal *che* operon (TK0629-TK0639) were significantly depleted. Additionally, a portion (TK0394-TK0410) of viral region 2 (TK0381-TK0421) was significantly depleted. **(C)** In TS622 (TK1413^*G*17*D*^:histone A/TK2289^*WT*^:histone B) far fewer genes were upregulated when compared to TS600. Downregulated genes related in cell motility and environmental signal sensing revealed similar trends to TS620 (*che* and *fla* operons).

RNAs from each strain were purified from cultures following the environmental shift, depleted of ribosomal RNAs, and subjected to RNA-sequencing to quantify steady-state transcript abundance. RNAs were collected at early time points following the environmental shift to monitor the primary impacts of variant AHCP architectures on gene expression. Differential expression analyses defined meaningful changes in the transcriptomes of TS620 and TS622 compared to TS600 (Figure 2). Comparison of transcriptomes resultant from TS600 (HTkA^*WT*^ only; typical AHCPs of varied lengths) and TS620 (HTkA^*G*17*D*^ only; containing only short AHCPs) provides a quantitative measure of the regulation normally afforded by AHCPs in *T. kodakarensis* (Figures 1C, 2). In contrast, comparison of the transcriptomes of TS600 and TS622 (HTkB^*WT*^, HTkA^*G*17*D*^) highlights the impact of histone variants on AHCPs that can regularly form with a mixture of HTkB and HTkA^*G*17*D*^ (Figures 1C, 2). Transcriptome changes were quantified by comparing log_2_-average fold change (Log_2_FC) against log-average counts per million (LogCPM) (Figures 2B,C). The *T. kodakarensis* genome encodes ∼2,300 annotated open reading frames, with abundant antisense transcription and many small transcripts (Fukui et al., 2005; Jäger et al., 2014; Sas-Chen et al., 2019). Our sequencing coverage was sufficient to detect nearly all transcripts with a LogCPM > 1.00: 2,225 transcripts were included in the TS620/TS600 comparison; 2,231 transcripts were included in the TS622/TS600 comparison.

The substitution of HTkA^*G*17*D*^ (TS620) for HTkA^*WT*^ (TS600) as the only histone in *T. kodakarensis* resulted in significant (> 2-fold) changes in the steady-state abundance of 234 genes, representing ∼11% of the entire transcriptome. The lack of extended AHCPs in TS620 resulted in increased abundance of 100 genes and decreased abundance of another 134 genes (Figure 2A); genes with both ordinarily high or low expression were differentially expressed (Jäger et al., 2014). Genes-encoding proteins involved in central metabolism, purine synthesis and metabolism, amino acid synthesis, transport and a number of hypothetical proteins were upregulated (Table 1A). No obvious chromosomal distribution was noted, with the obvious exception of coregulation of operons, suggesting the entire chromosome is normally subject to regulation imposed by extended AHCPs. Further, hinting that expression of histone-encoding genes are regulated by AHCPs, TK1413^*G*17*D*^ transcripts were enriched ∼2.8 fold in TS620 compared to TK1413^*WT*^ in TS600. Altered AHCP structures undoubtedly impact DNA replication, recombination and repair, and the loss of extended AHCPs results in the large increase in abundance of all three replication factor A proteins (archaeal RPA is a heterotrimer composed in *T. kodakarensis* of the products of TK1959 (increased ∼5.2 fold), TK1960 (increased ∼5.8 fold) and TK1961 (increased ∼4.8 fold) in strain TS620. The likely increased abundance of functional RPA proteins suggests inhibiting extended AHCPs may permit some DNA regions to locally unwind or melt, thereby requiring more RPA to protect the increased abundance of single-stranded DNA (Figure 2B).

**TABLE T1A:** Transcripts enriched and depleted in TS620 (TK1413^*G*17*D*^:histone A/ΔTK2289:histone B) compared to TS600 (TK1413^*WT*^:histone A/ΔTK2289:histone B). **(A)** The 30 most enriched transcripts in TS620 compared to TS600. **(B)** The 30 most depleted transcripts (*sans* viral region 2 transcripts) in TS620 compared to TS600.

A			

Transcript	Annotation	Process	Fold Change
TK1459	Hypothetical protein	Unknown	22.52
TK1358	Hypothetical protein	Unknown	21.63
TK2061	Sodium/phosphate symporter	Transport	12.84
TK0604	Hypothetical protein	Unknown, viral region 3	12.00
TK0605	Hypothetical protein	Unknown, viral region 3	9.75
TK0208	Phosphoribosyl formylglycinamidine cyclo-igase	Purine metabolism	9.51
TK0202	Phosphoribosyl formylglycinamidine ynthase, PurS component	Purine metabolism	9.18
TK0204	Phosphoribosylamine-glycine ligase	Purine metabolism	8.97
TK1392	NADH oxidase	Metabolsim	8.74
TK0203	Phosphoribosyl formylglycinamidine cyclo-ligase	Purine metabolism	8.43
TK1356	ATPase, AAA superfamily	Unknown, viral region 4	8.41
TK0835	Phosphoribosy laminoimidazole carboxylase, ATPase subunit	Purine metabolism	7 60
TK1393	Anaerobic glycerol 3-phosphate dehydrogenase	Lipid metabolism	7.40
TK2060	Distant homolog of phosphate transport system regulator PhoU	Transport	7.20
TK1391	Molybdopterin oxidoreductase, 4Fe-4S cluster-binding subunit	Central metabolsim	6.81
TK1023	Hypothetical protein	Unknown	6.64
TK0207	Format-dependent hosphoribosylglycinamide formyltransferase	Purine metabolism	6.42
TK1960	Replication factor A complex, RPA14 subunit	Replication/recombi nation/repair	6.42
TK0201	Phosphoribosy lformylglycinamidine synthase I	Purine metabolism	6.26
TK1464	Hypothetical protein	Unknown	6.05
TK0599	Hypothetical protein	Unknown, viral region 3	5.70
TK1959	Replication factor A complex, RPA32 subunit	Replication/recombi nation/repair	5.63
TK0389	Hypothetical protein	Unknown, viral region 2	5.57
TK0252	Indole-3-glycerol phosphate synthase	Amino acid synthesis	5.57
TK0836	Phosphoribosyl aminoimidazole carboxylase, catalytic subunit	Purine metabolism	5.50
TK0601	ATPase, AAA superfamily	Unknown, viral region 3	5.42
TK0580	Hypothetical protein	Unknown, viral region 3	5.39
TK0382	Hypothetical protein	Unknown, viral region 2	5.17
TK1961	Replication factor A complex, RPA41 ubunit	Replication/recombi nation/repair	5.14
TK0600	Hypothetical protein	Unknown, viral region 3	5.13

While many gene classes showed increases in transcript abundance due to changes in AHCP structure, a large percentage of genes related to cell motility and environmental signal sensing were downregulated in the absence of extended AHCPs in TS620 when compared to TS600. Localized AHCPs are likely to regulate gene expression both positively and negatively, depending on the availability of DNA sequences critical for gene expression. Expression of operons encoding archaellum components (annotated as the *fla* operon) and chemotaxis proteins (*che* operon) appear particularly sensitive to AHCPs and are likely dependent on AHCPs for proper regulation (Figure 2B). Genes comprising the entire *T. kodakarensis* archaellum-encoding *fla* operon (TK0038-TK0049) and chemotaxis-encoding *che* operon (TK0629-TK0639) were ∼7.1–65-fold and ∼2.7–10-fold less abundant in strain TS620 compared to strain TS600 (Table 1B).

**TABLE T1B:** 

**B**			

**Transcript**	**Annotation**	**Process**	**Fold change**
TK0038	Archaeal flagellin B1 precursor	Cell motility	−65.39
TK0039	Archaeal flagellin B2 precursor	Cell motility	−30.65
TK0040	Archaeal flagellin B3 precursor	Cell motility	−21.73
TK0042	Archaeal flagellin B5 precursor	Cell motility	−19.94
TK0041	Archaeal flagellin B4 precursor	Cell motility	−18.37
TK0043	Archaeal flagella-related protein C	Cell motility	−14.30
TK0044	Archaeal flagella-related protein D, internal insertion	Cell motility	−11.59
TK0045	Archaeal flagella-related protein F	Cell motility	−11.25
TK0046	Archaeal flagella-related protein G	Cell motility	−10.23
TK0632	Chemotaxis response regulator, CheY	Environmental information processing	−10.09
TK0631	Chemotaxis protein methyltransferase, CheR	Environmental information processing	−9.51
TK0634	Chemotaxis histidine kinase, CheA	Environmental information processing	−9.16
TK0633	Chemotaxis protein-glutamate methylesterase, containing receiver domain	Environmental information processing	−8.81
TK0635	Chemotaxis histidine kinase	Environmental information processing	−8.39
TK0156	Methyl-accepting chemotaxis protein	Environmental information processing	−8.34
TK2147	Methyl-accepting chemotaxis protein	Environmental information processing	−8.25
TK0049	Archaeal flagella-related membrane protein J	Cell motility	−8.09
TK0047	Archaeal flagella-related protein H	Cell motility	−7.39
TK0630	Methyl-accepting chemotaxis protein	Environmental information processing	−7.14
TK0048	Archaeal flagella-related protein I	Cell motility	−7.13
TK0637	Chemotaxis protein cheC	Environmental information processing	−6.89
TK0050	Hypothetical membrane protein	Unknown	−6.51
TK0636	Chemotaxis protein CheC	Environmental information processing	−6.36
TK0168	Predicted transcription regulator, Lrp/AsnC family	Transcription	−5.47
TK0546	Hypothetical protein	Unknown	−5.32
TK0638	Methyl-accepting chemotaxis protein	Environmental information processing	−5.21
TK1139	ATPase, AAA superfamily	Unknown	−5.19
TK1804	ABC-type dipeptide/oligopeptide transport system, probable periplasmic component	Transport	−4.50
TK1606	Methyl-accepting chemotaxis protein	Environmental information processing	−4.11
TK1605	Hydrolase, metallo-beta-lactamase superfamily	Unknown	−4.09

Given that AHCPs can be generated in strain TS622 – as assessed by MNase digestions (Figure 1) – we predicted a more minor impact on the total transcriptome of strain TS622 compared to TS600. Despite the presence of HTkA^*G*17*D*^, the added presence of HTkB in TS622 permits sufficient AHCP formation to reduce the number of aberrantly transcribed genes when compared to TS620. When comparing the transcriptomes of TS622 (HTkA^*G*17*D*^, HTkB) and TS600 (HTkA^*WT*^, ΔHTkB), we noted only approximately half as many transcripts (49) of decreased abundance in TS622, while just 18 transcripts were enriched (Figure 2C, Table 2A). Similar decreases in the abundance of transcripts encoding cell motility and environmental signal sensing were observed in TS622, with transcripts from the *fla-* and *che*-operon decreased ∼6–59-fold and ∼2.6–9-fold, respectively (Figure 2C, Table 2B). The decreased abundance of *fla-* and *che*-operon transcripts suggests the HTkA isoform is critical for proper regulation of these loci or factors that control expression of such loci and that the presence of HTkB^*WT*^ cannot compensate for the loss of HTkA^*WT*^ in these limited scenarios.

**TABLE T2A:** Transcripts enriched and depleted in TS622 (TK1413^*G*17*D*^:histone A/TK2289^*WT*^:histone B) compared to TS600 (TK1413^*WT*^:histone A/ΔTK2289:histone B). **(A)** All transcripts enriched in TS622 compared to TS600. Histone B, TK2289 denoted by * is absent in TS600 resulting in a large fold change. **(B)** The 30 most depleted transcripts in TS622 compared to TS600.

**A**			

**Transcript**	**Annotation**	**Process**	**Fold change**
TK2289	Archaeal histone B	Chromatin	1565.20
TK1020	Hypothetical membrane protein	Unknown	3.49
TK0717	Molybdate transport system substrate-binding protein	Transport	2.93
TK0162	Hypothetical membrane protein	Unknown	2.69
TK0718	Molybdate transport system permease protein	Transport	2.58
TK0720	Hypothetical protein	Unknown	2.55
TK0166	Hypothetical protein	Unknown	2.48
TK2070	Sulfhydrogenase subunit delta	Energy Uetabolism	2.45
TK1862	Hypothetical protein	Unknown	2.45
TK0467	Hypothetical protein	Unknown	2.42
TK2071	Sulfhydrogenase subunit gamma (sulfur reductase)	Energy metabolism	2.37
TK0719	Molybdate transport system ATP-binding protein	Transport	2.37
TK2072	Sulfhydrogenase subunit beta (sulfur reductase)	Energy metabolism	2.33
TK0164	S-layer-like array protein	Cell structure	2.33
TK0163	ABC-2 type transport system permease protein	Transport	2.27
TK1463	Hypothetical protein	Unknown	2.22
TK2278	Myo-inositol-1 -phosphate synthase	Metabolism	2.19
TK0765	Glyceraldehyde-3-phosphate dehydrogenase (NAD(P))	Metabolism	2.12
TK2069	Sulfhydrogenase subunit alpha	Energy metabolism	2.04

**TABLE T2B:** 

**B**			

**Transcript**	**Annotation**	**Process**	**Fold change**
TK0038	Archaeal flagellin B1 precursor	Cell motility	−59.54
TK0039	Archaeal flagellin B2 precursor	Cell motility	−41.95
TK0040	Archaeal flagellin B3 precursor	Cell motility	−30.60
TK0042	Archaeal flagellin B5 precursor	Cell motility	−29.13
TK0041	Archaeal flagellin B4 precursor	Cell motility	−25.02
TK0043	Archaeal flagella-related protein C	Cell motility	−17.47
TK0812	Adenylate kinase	Punne metabolism	−17.38
TK0811	Hypothetical protein	Unknown	−16.68
TK0631	Chemotaxis protein methyltransferase CheR	Environmental information processing	−13.03
TK0046	Archaeal flagella-related protein G	Cell motility	−9.90
TK0044	Archaeal flagella-related protein D, internal insertion	Cell motility	−9.76
TK0045	Archaeal flagella-related protein F	Cell motility	−9.40
TK0156	Methyl-accepting chemotaxis protein	Environmental information processing	−8.01
TK0635	Chemotaxis histidine kinase	Environmental information processing	−7.36
TK0632	Chemotaxis protein CheY	Environmental information processing	−7.27
TK0630	Methyl-accepting chemotaxis protein	Environmental information processing	−7.20
TK0048	Archaeal flagella-related protein I, predicted Csecretion ATPase	Cell motility	−6.92
TK0633	Chemotaxis protein-glutamate methylesterase, containing receiver domain	Environmental information processing	−6.89
TK0047	Archaeal flagella-related protein H, predicted ATPase	Cell motility	−6.71
TK0049	Archaeal flagella-related membrane protein J	Cell motility	−5.85
TK0634	Sensor kinase CheA	Environmental information processing	−5.83
TK0636	Chemotaxis protein CheC	Environmental information processing	−5.59
TK0050	Hypothetical protein	Unknown	−5.39
TK2147	Methyl-accepting chemotaxis protein	Environmental information processing	−5.19
TK0637	Chemotaxis protein CheC	Environmental information processing	−5.07
TK0431	5-formaminoimidazole-4-carboxamide-1-(beta)-D-ribofuranosyl 5’-monophosphate synthetase	Purine metabolism	−4.51
TK0638	Methyl-accepting chemotaxis protein	Environmental information processing	−4.43
TK0432	Phosphonbosylam inoimidazole-succinocarboxamide synthase	Purine metaboism	−4.26
TK1139	ATPase, AAA superfamily	unknown	−4.23
TK0051	Protein-L-isoaspartate(D-aspartate) *O*-methyltransferase	unknown	−4.01

### AHCPs Are Necessary for Proviral Region Expression and Retention

The *T. kodakarensis* genome contains 4 annotated, non-essential proviral regions, each ∼20–25 Kbp in length: TKV1 (TK0073-TK0105), TKV2 (TK0381-TK0421), TKV3 (TK0575-TK0614), and TKV4 (TK1342-TK1378) (Fukui et al., 2005; Tagashira et al., 2013). Comparisons of the genomes of many *Thermococcales* suggests recombination events between proviral regions can rearrange the genome context in evolutionary timescales; however, the proviral regions of the *T. kodakarensis* appear genetically stable (Krupovic et al., 2013; Cossu et al., 2015, 2017). These proviral regions represent ∼100 Kbp in total (∼5% of the 2.08 Mbp genome) and encode a plethora of genes with unknown function. Expression of the proviral regions has been observed in all transcriptome studies of *T. kodakarensis* (Tagashira et al., 2013; Jäger et al., 2014). An observed trend in the transcriptomic profiles obtained from strain TS620 was the depletion or enrichment of genes assigned to the *T. kodakarensis* viral regions (Figures 2B,D). Several predicted integrase genes (TK0104 and TK0381) were upregulated in TS620 as well as the non-essential PCNA2 (Pan et al., 2013) (TK0582) and a predicted AAA superfamily ATPase (TK0601), (Supplementary Figures 3A,B). Most intriguing, transcripts aligning to a central portion of TKV2 (TK0390, TK0394-TK0410) were completely depleted from TS620. Among these genes were several predicted SpoVT, AbrB transcriptional regulators, and many hypothetical genes (TK0402, TK0405, TK0406, TK0409, and from TKV4, TK1372).

**FIGURE 3:**
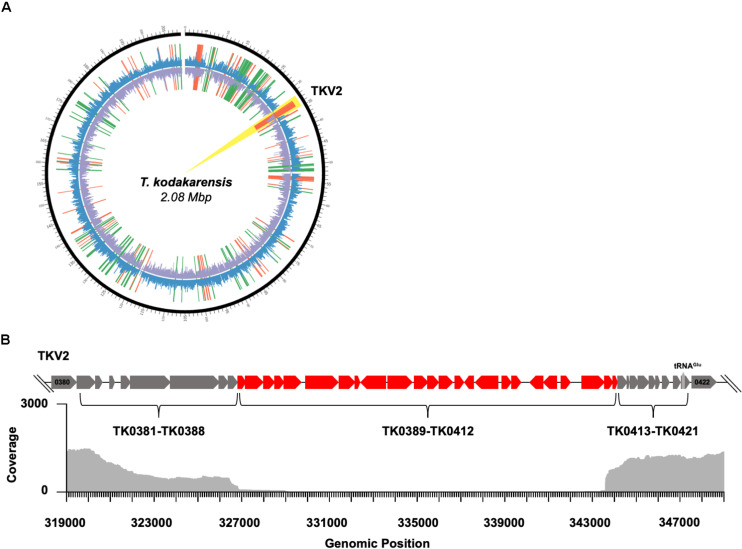
Disruption of 3-dimensional chromatin structure results in genome instability in *T. kodakarensis*. **(A)** A circos plot comparing TS620 to TS600. The outermost black circle represents genomic position. The outer coverage plot (blue) represents Fragments Per Kilobase of transcript per Million mapped reads (FPKM) for TS620. The inner coverage plot (purple) represents FPKM for TS600. Notably, nearly zero reads mapped to TKVR2 in TS620 (highlighted in yellow). Red lines represent fragments enriched in TS600 while green lines represent fragments enriched in TS620. **(B)** A loci diagram of the annotated *T. kodakarensis* viral region 2 (TKVR2: TK0381-TK0421) that highlights the observed region of excision (∼TK0389 – ∼TK0412) superimposed over a genome alignment plot derived from PacBio long read sequencing of TS620.

The observed complete absence of these TKV2 transcripts prompted further evaluation of the genome of TS620 (Figure 3). Whole-genome sequencing (WGS) of TS620 revealed a relatively large (∼15 Kbp) central region of TKV2 was spontaneously excised from the genome (Figure 3B). Excision of most, but not all of TKV2 was confirmed by PCR amplifications of loci within and flanking TKV2 sequences in the genome of TS620 (Supplementary Figure S2). This missing region of TKV2 within strain TS620 genomic sequences aligns closely with the observed lack of reads aligning to TKV2 (Figure 3A). Despite differential expression of portions of other viral regions, the genomic loci for TKVR1, TKVR3, and TKVR4 remain intact (Supplementary Figure S3). The excision of TKV2 in only TS620 suggests not only are AHCP necessary for regulated gene expression, but that these structures also play a role in genome stability and recombination, perhaps related to viral region retention or repression.

## Discussion

Histone proteins encoded in most Archaea are the primary proteins responsible for genome organization (Nalabothula et al., 2013; Peeters et al., 2015; Henneman et al., 2018; Henneman et al., 2020; Sanders et al., 2019b; Stevens et al., 2020). Despite geometric and structural similarities, the potential for archaeal histones to form a continuous helical polymer is distinct from the eukaryotic nucleosome (Mattiroli et al., 2017). Formation of AHCPs is a major regulatory event in adaptive gene expression in *T. kodakarensis* and the regulation afforded by AHCP structures likely extends to most histone-encoding Archaea (Bhattacharyya et al., 2018; Sanders et al., 2019b; Henneman et al., 2020; Stevens et al., 2020; Bowerman et al., 2021; Laursen et al., 2021).

The transcriptomes of *T. kodakarensis* strains that can (TS600 and TS622) and cannot generate extended AHCPs (TS620) reveal significant differences most likely associated directly with altered chromatin landscapes. The impacts are often dramatic, and the regulation is not associated with isolated chromosomal regions but instead is noted genome wide. In strain TS620, where formation of stable and detectable (via MNase digestions) AHCPs was inhibited, >11% of the transcriptome was significantly altered. Approximately equal numbers of transcripts were more or less abundant due to the loss of AHCPs, suggesting that AHCPs can both positively and negatively impact gene expression, depending on the loci. Expression of the *che- and fla*-operons, as well as many proviral regions were the most dramatically affected by changes in the AHCP formation. In support of AHCP length influencing gene expression in these operons/proviral regions, expression profiles of *T. kodakarensis* strains encoding WT versions of a single histone isoform displayed no significant differences in the expression of these same operons (Čuboňová et al., 2012). AHCPs composed entirely of HTkA or HTkB are known to be sufficient for normal expression suggesting the presence of HTkA^*G*17*D*^ disrupts AHCP formation, thereby altering transcription in both TS620 and TS622. It is also plausible disrupted chromatin structures limit or increase expression of transcription factors that regulated select operon expression, but our transcriptomics data does not identify any obvious candidates.

Perhaps the most striking dysregulation observed in strains incapable of chromatin superhelix formation (TS620) was the loss of a portion of TKV2 from the genome. Like many proviral integrations in archaeal genomes, all four proviral regions in the *T. kodakarensis* genome overlap with tRNA encoding loci and large rearrangements noted in the genomes of *T. kodakarensis* and related *Thermococcales* often begin and end internal to the proviral regions (Geslin et al., 2007; Ortmann et al., 2008; Soler et al., 2008; Gonnet et al., 2011; Mochizuki et al., 2011; Gorlas et al., 2012; Tagashira et al., 2013). The combined impacts of disrupted AHCP formation on replication and recombination, the increased abundance of predicted viral integrase transcripts, and the potential for more ssDNA due to DNA melting in strains with altered chromatin landscapes provides a plausible explanation for the loss of TKV2 sequences. The inability to detect DNA sequences encompassing TK0389-0412 or viral particles containing these genes suggests this DNA fragment was degraded following excision from the genome (Soler et al., 2008). Although dispensable, the obvious growth defects of *T. kodakarensis* strains lacking proviral regions suggest their incorporation and proper regulation within archaeal genomes confers an evolutionary advantage (Hawkins et al., 2013; Tagashira et al., 2013).

The retention of histone proteins in most archaeal clades suggest histone-based chromatin structures provide beneficial regulatory roles that are exploited to provide a level of regulation on gene expression. The varied lengths of AHCPs, the presence of multiple histone isoforms in many Archaea, and the known changes in histone isoform expression in response to environmental changes all present routes to activate, repress and fine-tune gene expression to maximize growth in changing environments (Mattiroli et al., 2017; Bhattacharyya et al., 2018; Henneman et al., 2018; Henneman et al., 2020; Sanders et al., 2019b; Stevens et al., 2020). The potential for biologically significant post-translational modification of archaeal histones may provide an additional regulatory avenue (Alpha-Bazin et al., 2020).

The mechanisms controlling the formation of AHCPs with varying lengths at different loci and in different sequence contexts likely plays an additional role in transcription regulation (Henneman et al., 2018; Henneman et al., 2020; Sanders et al., 2019a,b, 2020; Stevens et al., 2020) and may be exploited by yet to be discovered archaeal chromatin remodeling complexes or histone isoforms that promote or inhibit formation of archaeal chromatin superstructures.

## Data Availability Statement

The datasets presented in this study have been deposited in the NIH GEO repository at https://www.ncbi.nlm.nig.gov and can be accessed with accession number GSE151920.

## Author Contributions

TSd, SF, RV, BB, and TSt designed, constructed, and confirmed the local genotypes of *T. kodakarensis* strains used in this work. TSd purified proteins, DNAs and RNAs, performed MNase digestions, generated RNA-seq libraries, analyzed data, built structural models, prepared figures, and helped write the manuscript. FU and AB-H analyzed RNA-sequencing datasets, prepared figures and helped write the manuscript. AGe and AGa prepared and sequenced DNA sequencing libraries, analyzed whole genome sequencing results, prepared figures, and helped write the manuscript. TSd and TSt prepared the initial manuscript that was edited, improved, and ultimately approved by all authors. All authors contributed to the article and approved the submitted version.

## Conflict of Interest

AGe and AGa are employed and funded by New England Biolabs, Inc. New England Biolabs is a manufacturer and vendor of molecular biology reagents, including DNA replication and repair enzymes. This affiliation does not affect the authors’ impartiality, objectivity of data generation or its interpretation, adherence to journal standards and policies or availability of data. The remaining authors declare that the research was conducted in the absence of any commercial or financial relationships that could be construed as a potential conflict of interest.

## Supplementary Material

Supplementary Figure 1 Construction of TS600, TS620, and TS622. **(A)** Genomic loci diagrams for histone A (TK1413) and histone B (TK2289) and the modifications to each in strains TS600, TS620, and TS622 compared to parental strain TS559. **(B)** Amplification of TK1413 and TK2289 using the primers diagramed in **(A)**. The open reading frame for TK2289 has been markerlessly deleted in both TS600 and TS620. Amplicons for TK1413 (lanes 1, 2, and 3) were sequence and **(C)** confirmed to contain either a glycine codon at position 17 (TS600) or aspartic acid (TS620 and TS622).

Supplementary Figure 2 Viral region expression dysregulation results in the partial loss of TKV2. **(A)** All viral region transcripts enriched in TS620 when compared to TS600. **(B)** All viral region transcripts depleted in TS620 when compared to TS600. **(C)** Primer Pairs A/B, C/D, and E/F were used to amplify the upstream, downstream and central region of TKV2 in 1-TS600, 2-TS622, and 3-TS620.

Supplementary Figure 3 TKV1, TKV3, and TKV4 remain stably integrated in the *T. kodakarensis* genome. TKV1 spans TK0073-TK0105. TKV3 spans TK0575-TK0614. TKV4 spans TK1342-TK1378.
