# Supplementary material for: Extended Archaeal Histone-Based Chromatin Structure Regulates Global Gene Expression in Thermococcus kodakarensis
Source: Front Microbiol. 2021 May 13;12:681150. doi: 10.3389/fmicb.2021.681150 (PMC8155482; doi:10.3389/fmicb.2021.681150)
Supplement: Supplementary Figure 1 — Construction of TS600, TS620, and TS622. (A) Genomic loci diagrams for histone A (TK1413) and histone B (TK2289) and the modifications to each in strains TS600, TS620, and TS622 compared to parental strain TS559. (B) Amplification of TK1413 and TK2289 using the primers diagramed in (A). The open reading frame for TK2289 has been markerlessly deleted in both TS600 and TS620. Amplicons for TK1413 (lanes 1, 2, and 3) were sequence and (C) confirmed to contain either a glycine codon at position 17 (TS600) or aspartic acid (TS620 and TS622). [file Data_Sheet_1.docx]

**SUPPLEMENTARY INFORMATION**

**Extended archaeal histone-based chromatin structure regulates global gene expression in *Thermococcus kodakarensis*.**

Travis J. Sanders^1^, Fahad Ullah^2^, Alexandra M. Gehring^3#^, Brett W. Burkhart^1^, Robert L. Vickerman^1^, Sudili Fernando^1^, Andrew F. Gardner^3^, Asa Ben-Hur^2^ and Thomas J. Santangelo^1^*

1 - Department of Biochemistry and Molecular Biology, Colorado State University, Fort Collins, Colorado, 80523, USA

2 – Department of Computer Science, Colorado State University, Fort Collins, Colorado, 80523, USA

3 – Molecular Enzymology Division, New England Biolabs, Inc., Ipswich, Massachusetts, 01938, USA

*Correspondence: [thomas.santangelo@colostate.edu](mailto:thomas.santangelo@colostate.edu)

Phone: +1-970-491-3150

^#^Current Address: Arrakis Therapeutics, Waltham, Massachusetts 02451, USA

Running title: Archaeal chromatin regulates gene expression

**Supplementary Figure 1**


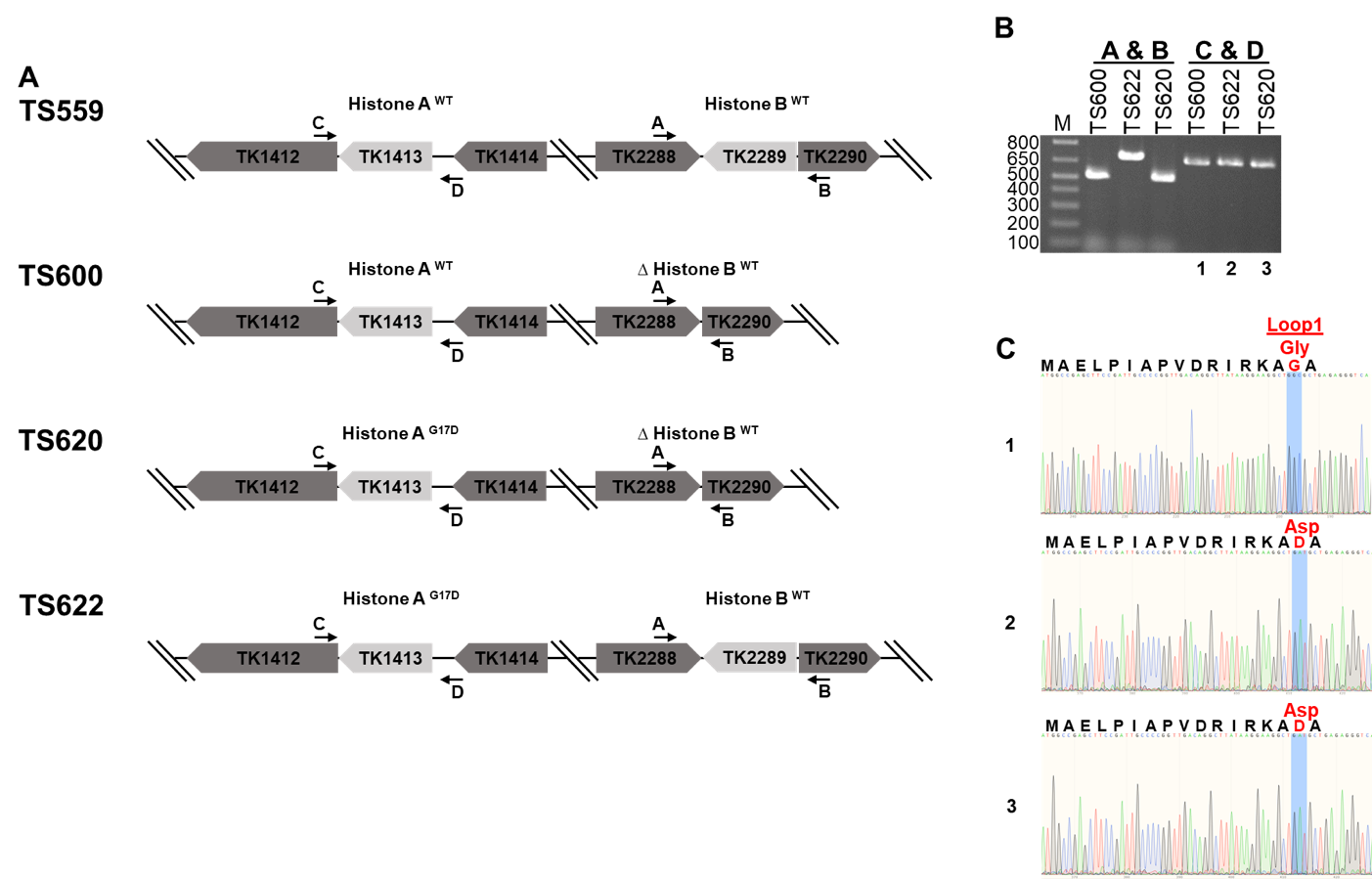


**Construction of TS600, TS620, and TS622. A)** Genomic loci diagrams for histone A (TK1413) and histone B (TK2289) and the modifications to each in strains TS600, TS620, and TS622 compared to parental strain TS559. **B)** Amplification of TK1413 and TK2289 using the primers diagramed in (A). The open reading frame for TK2289 has been markerlessly deleted in both TS600 and TS620. Amplicons for TK1413 (lanes 1, 2, and 3) were sequence and **C)** confirmed to contain either a glycine codon at position 17 (TS600) or aspartic acid (TS620 and TS622).

**Supplementary Figure 2**

**A**

**
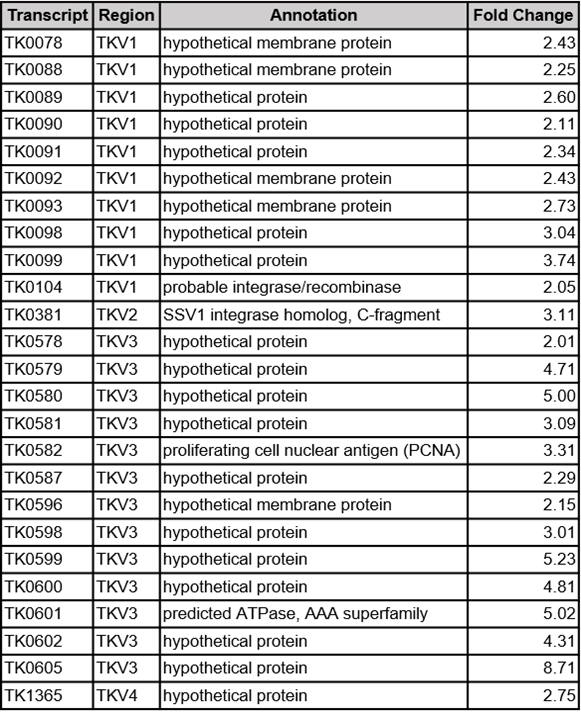
**

**B**

**
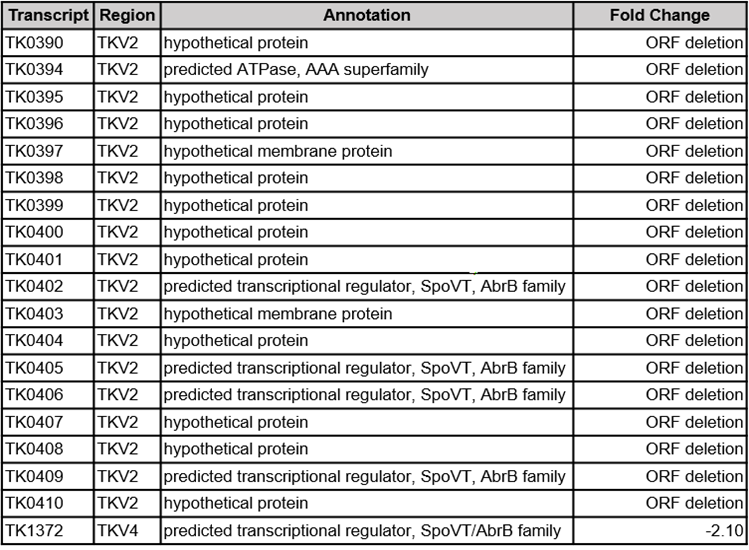
**

**
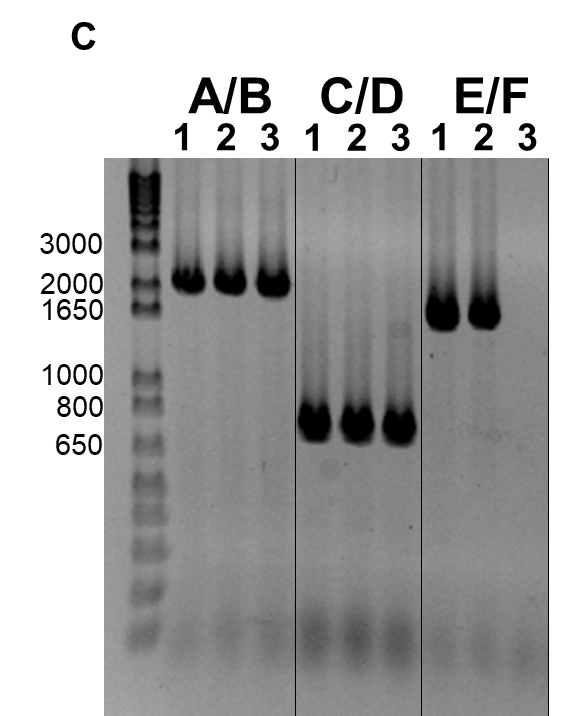
**

**
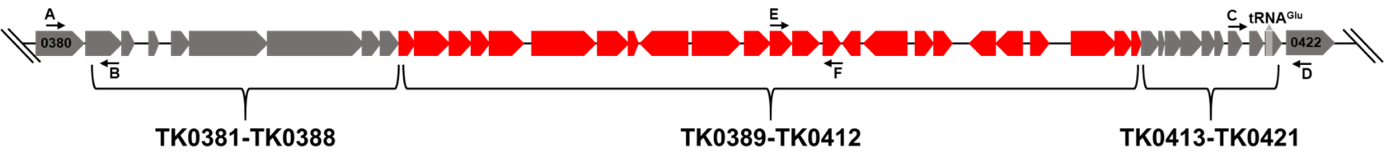
**

**Viral region expression dysregulation results in the partial loss of TKV2. A)** All viral region transcripts enriched in TS620 when compared to TS600. **B)** All viral region transcripts depleted in TS620 when compared to TS600. **C)** Primer Pairs A/B, C/D and E/F were used to amplify the upstream, downstream and central region of TKV2 in 1-TS600, 2-TS622 and 3-TS620.

**
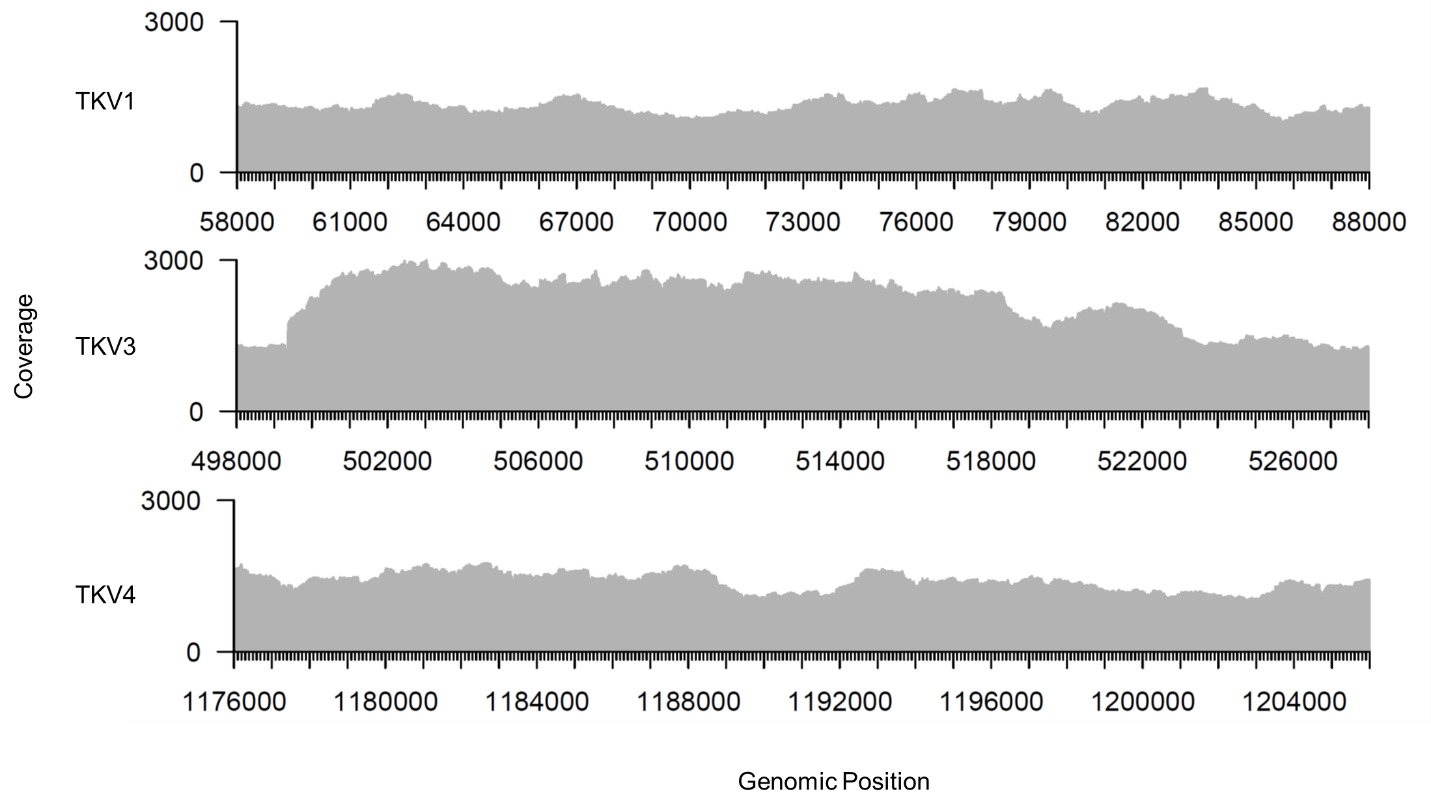
Supplementary Figure 3**

**TKV1, TKV3, and TKV4 remain stably integrated in the *T. kodakarensis* genome.** TKV1 spans TK0073-TK0105. TKV3 spans TK0575-TK0614. TKV4 spans TK1342-TK1378.
